# The Relation of Mood and Sexual Desire: An Experience Sampling Perspective on the Dual Control Model

**DOI:** 10.1007/s10508-022-02357-w

**Published:** 2022-07-27

**Authors:** Piet van Tuijl, Peter Verboon, Jacques van Lankveld

**Affiliations:** 1 Department of Psychology, Open Universiteit, 6419 Heerlen, The Netherlands; 2 Egelantierstraat 138, 3551GG Utrecht, Netherlands

**Keywords:** Sexual motivation, Positive and negative affect, Dual control model, Inertia, Experience sampling method

## Abstract

We investigated the effect of fluctuations in negative and positive affect on momentary sexual motivation in a sample of women and men in a steady relationship (*n* = 133). Sexual motivation was regarded as the aggregate of sexual desire, subjective sexual arousal and openness to sexual contact. Experience sampling methodology was used to collect up to 70 measurements per participant over a period of seven consecutive days of sexual motivation, and negative and positive affect. Using multilevel analysis, we investigated cross-level interactions between affect and trait measures as specified in the dual control model (DCM). This model postulates sexually excitatory and inhibitory mechanisms as relatively independent systems that together can explain individual differences in sexual motivation and behavior. Results implicated that any intensification of feelings, positive or negative, was associated with a momentary increase in sexual motivation for participants more prone to sexual excitation. In the lagged analysis, higher preceding negative affect, measured 1–2 h earlier, forecasted an increase in current sexual motivation for participants more prone to sexual excitation. The lagged analysis included the autoregressive effect or inertia of sexual motivation. Inertia reflects the extent to which sexual motivation lingers and persists at similar levels. Our findings showed that sexual motivation levels persisted less in individuals with higher sexual inhibition proneness due to threat of performance failure. This study demonstrated how experience sampling methodology can be used to extend research on associations between mood and sexual motivation and implicates that DCM factors moderate these associations.

**Supplementary Information:**

The online version contains supplementary material available at 10.1007/s10508-022-02357-w.

## Introduction

Momentary sexual motivation, regarded as the moment-to-moment experience of one’s motivation to become sexual, has often been associated with certain mood states (Janssen et al., 2013; Lykins et al., 2006). The commonly used phrase “getting in the mood for sex” expresses there might be specific mood states that induce the motivation to become sexual. The impact of mood on sexual motivation has usually been investigated in cross-sectional and experimental studies and results suggest temporal patterns in which mood impacts sexual motivation (Bancroft et al., 2003a; Peterson & Janssen, 2007; Ter Kuile et al., 2009). However, methodologically, cross-sectional and experimental research might be less suited to investigate associations between mood and sexual motivation as these methods cannot take the fluctuating nature of momentary sexual motivation fully into account. Longitudinal research into fluctuations in sexual motivation often investigated between-day fluctuations, using daily diary methods (e.g., Dewitte & Mayer, 2018; Kalmbach & Pillai, 2014; Muise et al., 2019). However, sexual motivation can fluctuate at shorter intervals, as shown by a few studies (Mehta et al., 2013; Miner et al., 2019; Van Lankveld et al., 2018). Moreover, mood states can change considerably over the course of a day (e.g., Koval & Kuppens, 2012). Therefore, we applied an intensive longitudinal design (ILD) in the current study to investigate fluctuations in mood and sexual motivation. The experience sampling strategy of ILD involves multiple measurements per participant per day over several consecutive days (Csikszentmihalyi, 2014). By using ILD, we can investigate the trajectories of mood and sexual motivation throughout the day and assess if mood fluctuations impact momentary sexual motivation.

Momentary sexual motivation, conceptualized as the composite of momentary sexual desire, sexual arousal and openness to sex, represents feelings that—to a greater or lesser extent—stem from sexual fantasies, urges and behavior. Of its components, sexual desire can be described as the amalgam of feelings that incline us to behave sexually (Levine, 2003). Sexual desire is characterized by large differences in intensity and manifestation (Toates, 2014) with large variation in the object of desire. Subjective sexual arousal denotes how the individual experiences her or himself as sexually aroused, which might not be fully in accordance with genital sexual arousal (Chivers et al., 2010; Laan et al., 1995). Subjective sexual arousal is not always distinguishable from sexual desire as indicated by both women (Graham et al., 2004) and men (Janssen et al., 2007) participating in focus group studies. For some participants of these studies, sexual desire could precede but also follow arousal (Graham et al., 2004). Openness to sex can be described as interest in the opportunity of sexual activity, might it present itself, and can thus be considered as a more passive component of sexual motivation. In combination, fluctuations in sexual desire, subjective sexual arousal and openness to sex express momentary sexual motivation. As such, it can be investigated as a part and consequent of emotion regulation processes (Everaerd et al., 2006).

The expression “to be in the mood,” commonly used to describe the desire for sex or the experience of oneself as sexual aroused, suggests a well-determinable mood state that accompanies higher levels of sexual motivation. Nonetheless, when investigating the impact of mood states on sexual interest, large individual differences have been found (Bancroft et al., 2003a; Janssen et al., 2013). Apparently, “to be in the mood” means different things to different people. Cross-sectional research has shown that positive mood in general is associated with an increase in sexual desire, although for some individuals much less than for others (Janssen et al., 2013; Nimbi et al., 2019). Negative mood is commonly established as a factor that dampens sexual desire (Graham et al., 2004; Janssen et al., 2007; Mehrabian & Stanton-Mohr, 1985). However, some participants from interview studies reported that negative mood states, such as frustration or stress, can also increase sexual desire or arousal (Graham et al., 2004), while another interviewee mentioned that “mood don’t usually make a difference” (Janssen et al., 2007, p. 259). Importantly, large-scale cross-sectional research has shown that for a significant minority of the population, sexual interest increases when feeling depressed or anxious, while for a majority sexual interest typically decreases in such states (Bancroft et al., 2003a, 2003b; Lykins et al., 2006). These combined findings demonstrate the large variability in mood states that can impact sexual motivation.

In line with cross-sectional studies, experimental research has consistently shown that higher positive affect is related to higher sexual desire, while results regarding negative affect have been more equivocal (Peterson & Janssen, 2007; Ter Kuile et al., 2009). Ter Kuile et al. (2009) found that after sad mood induction, lower subjective sexual arousal levels were reported than after happy mood induction. However, Peterson and Janssen (2007) reported specific experimental conditions in which ambivalent affect, the combination of high positive and high negative affect, was positively correlated with sexual desire. They suggested that highly emotional content, both positive and negative, was predictive of sexual desire in these conditions. The equivocal results concerning negative affect might be in line with the considerable individual differences in associations between negative mood and sexual interest, reported in cross-sectional research (Bancroft et al., 2003a). In experimental research, randomly constructed groups are compared; if these groups are heterogeneous samples from the population, the effect of negative mood on sexual desire will be heterogeneous as well, leading to diverging results for negative mood. The equivocal results regarding negative affect, together with the substantial variability found in cross-sectional research for both positive and negative affect impacting sexual interest and desire, suggest that relevant moderators should be included in research into the associations between mood states and momentary sexual motivation (Peterson & Janssen, 2007).

The relation between mood and aspects of sexual motivation might be impacted by factors of the dual control model (DCM; Bancroft, 1999; Bancroft & Janssen, 2000). The DCM postulates “that individuals vary in their propensity for both sexual excitation and inhibition of sexual response” (Bancroft et al., 2003a, p. 218). Within the DCM, three aspects are discerned: sexual excitation proneness (SES), sexual inhibition proneness due to threat of performance failure (SIS1) and sexual inhibition proneness due to threat of performance consequences (SIS2). A positive association between SES and sexual interest in negative mood states was found in several studies (Bancroft & Vukadinovic, 2004; Bancroft et al., 2003a), implicating that people with higher sexual excitation proneness are more likely to show sexual interest in negative mood states. Negative associations between SIS1 and SIS2 and sexual interest in negative mood states were found in general population samples (Bancroft et al., 2003a; Lykins et al., 2006), although not in a self-identified sex addiction sample (Bancroft & Vukadinovic, 2004). These results suggest that the DCM might offer viable moderators for the relation between mood and momentary sexual motivation. After initial studies into the relation between mood, sexual interest and the three factors of the DCM (Bancroft & Vukadinovic, 2004; Bancroft et al., 2003a, 2003b; Lykins et al., 2006), such studies have not been conducted anymore as far as we know.

**Fig. 1:**
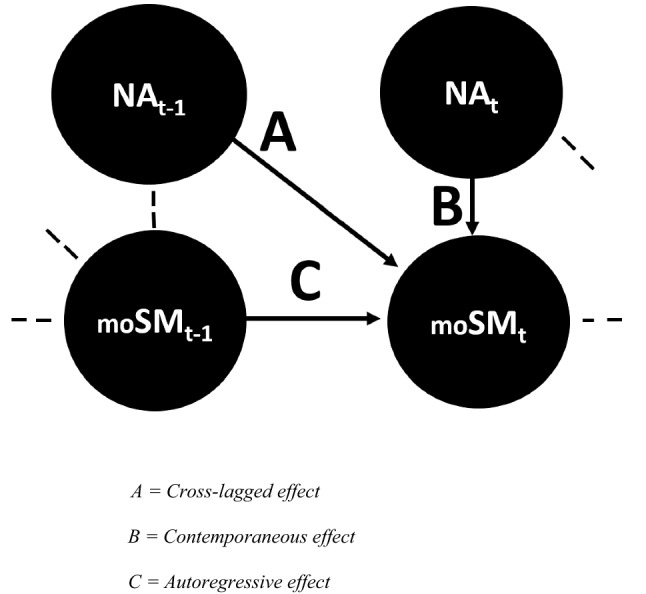
Conceptual model for the analyses of the time series of negative affect (NA) and momentary sexual motivation (moSM)

Common to previous cross-sectional and experimental research on mood and sexuality is the suggestion of temporal associations between mood states and momentary sexual motivation. However, such temporal associations might be better investigated with an ILD, because it enables assessment of the actual trajectories in time of mood and sexual motivation. ILD allows for the ecologically valid investigation (Verhagen et al., 2016) of intraindividual processes while simultaneously accounting for interindividual differences in these processes (Kuppens & Verduyn, 2017). The patterns underlying the fluctuating trajectories can be uncovered by applying the conceptual model presented in Fig. 1. Note that we present negative affect in Fig. 1 but the same model applies to other time-varying variables as well.

The model in Fig. 1 represents how current sexual motivation at time point t is predicted by three separate variables. First, current sexual motivation is predicted by negative affect measured at the preceding measurement, time point *t* – 1. This is a cross-lagged effect (‘A’ in Fig. 1). Second, current sexual motivation is predicted by negative affect measured at the current measurement, time point *t*. This is a contemporaneous effect (‘B’ in Fig. 1). Third, current sexual motivation is predicted by sexual motivation measured at the preceding measurement, time point *t* – 1. This is the autoregressive effect (‘C’ in Fig. 1). For the autoregressive and cross-lagged effects, the time lag between measurements importantly impacts the interpretation. Most often a time lag of 90 min is applied in ILD research to investigate short-term fluctuations in emotions and feelings.

The inclusion of the autoregressive effect (effect “C” in Fig. 1) allows for the investigation of the “inertia” (Suls et al., 1998, p.134) of sexual motivation. Inertia, seen as a person-level trait, expresses to what extent feelings or emotions linger and are resistant to change. It has been shown, in particular for positive and negative affect, that higher levels of short-term inertia are associated with maladaptive person-level characteristics such as depression, anticipatory stress and neuroticism (Koval & Kuppens, 2012; Kuppens et al., 2010; Suls et al., 1998). Higher inertia of affect implies that affect will fluctuate less throughout the day, and thus, the individual will be less capable to adapt to changing emotional circumstances (Kuppens & Verduyn, 2017). It is not clear if the short-term inertia of sexual motivation behaves in a similar way as the inertia of affective states. To the best of our knowledge, no studies have been published on the short-term inertia of sexual motivation and its associations. Only one study we know of mentioned an inertia value for sexual desire, but in that case as a control variable in a daily diary study (Kalmbach & Pillai, 2014) with a time lag of 24 h between measurements.

Methodologically, it is expedient to include the inertia effect because, in time series analyses, the preceding state is often the most important predictor of the current state, and leaving the inertia effect out might lead to spurious results (De Haan-Rietdijk et al., 2016). Additionally, including the inertia or autoregressive effect allows for conclusions about Granger causality (Granger, 1969): if there is a cross-lagged effect (A in Fig. 1) in a model in which the autoregressive effect is included as well (C in Fig. 1) than the predictor (e.g., lagged negative affect) is said to Granger cause, or forecast, the outcome (e.g., current sexual motivation). Granger causality is not equivalent to absolute causality (Granger, 1969) as there might be other explanatory variables that have not been included in the analyses.

There are a few ILD studies on positive and negative mood states and sexual desire or subjective sexual arousal, but none of these include the short-term inertia that is investigated in the current study. Moreover, previous ILD studies on mood and sexuality did not investigate moderators suggested by theory (such as the DCM). Mehta et al. (2013), using only analyses of contemporaneous effects, showed that positive affect was a better predictor of sexual desire than negative affect. Miner et al. (2019), using only lagged analyses, showed that the combination of higher positive and higher negative affect increased the probability of masturbating or using pornography at a later moment, in particular for participants who self-identified as being addicted to sex. Kalmbach and Pillai (2014), including the inertia of sexual desire and subjective sexual arousal as control variables, showed that after a relatively extended time lag of 24 h positive inertia effects were still present for desire and subjective sexual arousal. They also found positive contemporaneous and lagged associations between feeling happy and sexual desire. However, with a time lag of 24 h, aspects of lagged negative affect did not significantly impact sexual desire or arousal anymore (Kalmbach & Pillai, 2014). These studies illustrate that both contemporaneous and lagged analyses can provide meaningful insights into the associations between mood states and momentary sexual motivation.

### The Present Study

In the present study, we investigated the dynamic interplay between negative and positive mood states and momentary sexual motivation using experience sampling methodology. We used an intensive longitudinal design (ILD), which allows for the investigation of interindividual differences in within-person fluctuations of mood and sexual motivation. We analyzed both contemporaneous and temporal associations, and, importantly, also included the inertia effect of sexual motivation. Outcomes of this study were expected to provide insight into the associations between positive and negative mood states and momentary sexual motivation throughout the day. Also, we expected that outcomes would provide information about individual variability in the associations between mood and sexual motivation, and whether these associations were moderated by trait-like characteristics, specifically by sexual excitation and sexual inhibition proneness as conceptualized within the DCM framework.

#### Hypotheses

Associations between fluctuations in mood and momentary sexual motivation.

We hypothesized that higher negative affect would be associated with lower momentary sexual motivation, and that higher positive affect would be associated with higher momentary sexual motivation, both for the contemporaneous and the lagged analyses. For both effects, we expected that there would be considerable individual variability, in line with previous results of cross-sectional research (Bancroft et al., 2003a, 2003b; Janssen et al., 2013; Lykins et al., 2006).

### Inertia of Sexual Motivation

We hypothesized that the relation between current sexual motivation and the preceding level of sexual motivation would be positive, given an average time lag of 90 min. This means that we expected that inertia values of sexual motivation would be positive but not larger than 0.6, as is typical for constructs based on self-reports of emotions and feelings (Hamaker & Grasman, 2015). We also expected that the inertia value would not be smaller than 0.12, which is the sexual desire inertia value reported for daily diary data with a time lag of 24 h (Kalmbach & Pillai, 2014). The inertia value is lag dependent with smaller time lags usually resulting in larger inertia values.

### Moderation Effects of the Three Factors of the Dual Control Model

We hypothesized that sexual excitation proneness (SES) would moderate the association between mood and momentary sexual motivation and we expected similar effects for the contemporaneous and the lagged analyses. Although previous research (Bancroft et al., 2003a) showed that an increase in sexual interest when anxious or depressed occurred in people more prone to sexual excitation, we did not expect this effect in the current sample due to the higher mean age of the current sample and the fact that most participants were involved in long-term relationships. However, we did expect a weaker negative association between contemporaneous or lagged negative affect and momentary sexual motivation in participants with higher SES levels. Furthermore, we expected a stronger positive association between contemporaneous or lagged positive affect and momentary sexual motivation in those with higher SES levels.

We hypothesized that sexual inhibition proneness, due to threat of performance failure (SIS1), and sexual inhibition proneness, due to threat of performance consequences (SIS2), would moderate the association between mood and momentary sexual motivation. We expected a stronger negative association between contemporaneous or lagged negative affect and sexual motivation in those with higher SIS1 or SIS2 levels, in line with previous results (Bancroft et al., 2003a, 2003b; Lykins et al., 2006). We expected a weaker positive association between contemporaneous or lagged positive affect and sexual motivation for individuals with higher SIS1 and SIS2 levels.

With regard to the inertia of sexual motivation, we explored the moderation effect of SES, SIS1 and SIS2. No hypotheses were formulated regarding this moderation effect, due to a lack of previous research.

## Method

### Sample and Procedure

For this sample, participants were recruited by using snowball sampling via the personal contacts of seven master students. Informed consent forms were read and signed. Participants did not receive any monetary compensation. Inclusion criteria were: (1) engaged in a romantic heterosexual relationship of at least 6 months; (2) older than 17 years; (3) having at least 8 years of education, signifying completion of primary education (in order to secure comprehension of the questionnaires); and (4) speaking Dutch. Although only participants involved in heterosexual relationships were recruited, 4 participants (3%) indicated to be exclusively homosexual. Differences between hetero- and non-heterosexual participants were not further investigated as the number of non-heterosexual participants was too small to allow for meaningful group comparison. Of 133 participants, 87 (65.4%) were women with a mean age of 39.3 years (SD = 10.7), an average relationship duration of 13.4 years (SD = 9.7) and an average of completed years of education of 13.6 (SD = 2.4). The mean age of the 46 male participants (34.6%) was 46.4 years (SD = 11.4), their average relationship duration was 16.9 years (SD = 13.0), and they completed on average 13.5 years of education (SD = 3.1). Of the women 89% identified as Caucasian; of the men 96%. Only one couple participated in the study. The data were collected in 2012–2014 and ethical approval for the study was obtained from the institutional review board of Open University, the Netherlands. The sample used in the current study has been reported on previously by Van Lankveld et al. (2018) in a study focusing on the association between lagged intimacy and sexual desire. In that study, data were also collected on sexual excitation, sexual inhibition and positive and negative mood states, but these variables have until now not been used for research.

The start-up questionnaire, assessing most of the time-invariant trait characteristics of participants, was completed before the start of the experience sampling data collection. Experience sampling data were collected as follows: for 7 consecutive days and 10 moments per day participants were asked to complete a short survey: the beep questionnaire. Participants received a wristwatch and seven diary booklets with in each booklet 10 duplicates of the same survey. Ten time windows of 90 min between 7:30 and 22:30 were constructed and at random time points within each window, a signal sounded to prompt completion of the paper-and-pencil beep survey. The questionnaire consisted of approximately 40 short questions measuring the participant’s mood, sexual motivation and sexual activity and took 2–3 min to complete, depending on routine and the ability of the participant to respond “without thinking.” A maximum time period of 15 min was set for respondents to react to the signal. When participants completed less than 24 surveys out of a maximum of 70 (Delespaul, 1995), their data were excluded from the analyses.

### Time-Varying Variables

#### Momentary Sexual Motivation

Momentary sexual motivation was measured at beep level with 3 items using 7-point Likert-type scales. Items were worded “I feel sexually aroused,” “I feel sexual desire” and “I am open to sexual initiative.” For each person and measurement moment, the scores on the 3 items were averaged to calculate the sexual motivation score for that person at that specific moment. Multilevel confirmatory factor analysis on the full set of 14 beep-level items on sexual motivation (3 items), positive mood (5 items) and negative mood (6 items) was performed using the “lavaan” package (Rosseel, 2012) within the R environment (R Core team, 2021). An a priori specified three-factor model for the total of 14 beep-level items fitted the data well and confirmed momentary sexual motivation, positive and negative affect as three separate factors. For the construction and specific items used for positive and negative affect, see the description of these variables below. The fit measures for the factor analysis were: Comparative Fit Index (CFI) = 0.96, Tucker Lewis Index (TLI) = 0.95, root mean square error of approximation (RSMEA) = 0.04, standardized root mean square residual (SRMR) = 0.05 (within) and 0.07 (between). Factor loadings are reported in Table 1 in the supplementary material. Momentary sexual motivation showed adequate reliability, with McDonald’s ω (1999) of 0.86 at the person level and 0.72 at the beep level, as measured using the R-package “multilevelTools” (Wiley, 2020). We furthermore found strong positive correlations between the three items of sexual motivation with correlations varying between 0.76 and 0.85. This implicates that for this study sexual desire, subjective sexual arousal and openness to sex can be aggregated as a single construct. These results are in line with the qualitative findings of Graham et al. (2004) that showed that often sexual desire and subjective sexual arousal are difficult to distinguish in everyday experience.

**Table 1:** Means and standard deviations for women and men, with* p* values for* t* tests and effect sizes for the difference between women and men

	Women	Men	All		
	*M* (SD)	*M* (SD)	*M* (SD)	*p* value *t* test	Cohen’s d [95% CI]
Time-varying variables					
Momentary sexual motivation^a^	1.69 (0.80)	2.33 (1.03)	1.91 (0.93)	** < .001**	−.72 [−1.09 to −0.35]
Inertia sexual motivation^b^	0.38 (0.31)	0.44 (0.34)	0.40 (0.32)	.36	−.18 [−.54 to 0.19]
Negative affect^a^	1.25 (0.26)	1.28 (0.31)	1.26 (0.28)	.69	−.08 [−.44 to 0.28]
Positive affect^a^	4.93 (0.82)	5.25 (0.65)	5.04 (0.78)	**.01**	−.43 [−.80 to −.07]
Person level variables					
Age (years)	39.3 (10.7)	46.4 (11.4)	41.7 (11.4)	**.001**	−.64 [−1.01 to −.27]
Relationship duration (years)	13.4 (9.7)	16.9 (13.0)	14.6 (11.0)	.12	.32 [−.68 to 0.05]
SES	14.41 (3.37)	16.83 (2.86)	15.25 (3.39)	** < .001**	−.76 [−1.13 to −.38]
SIS1	10.38 (2.03)	9.13 (2.10)	9.95 (2.13)	**.001**	0.61 [0.24 to 0.98]
SIS2	11.95 (2.37)	10.70 (2.55)	11.52 (2.50)	**.007**	0.52 [0.15 to 0.89]
Depressive symptoms	19.24 (5.94)	17.43 (4.88)	18.62 (5.65)	.06	0.33 [−.04 to 0.68]
Anxiety	12.73 (2.75)	12.45 (3.61)	12.63 (3.07)	.65	0.09 [−.27 to 0.45]
Sexual frequency (p/week)	1.93 (1.98)	2.22 (2.11)	2.03 (2.02)	.45	0.14 [−.22 to 0.50]

Bold values are statistically significant (*p* < .05)

^a^To assess the means of the time-varying variables, scores were aggregated at a person-level average

^b^To assess individual’s inertia of sexual motivation, separate regression analyses for each participant were used with lagged sexual motivation as predictor of current sexual motivation

#### Lagged Sexual Motivation

In order to predict current sexual motivation by the preceding levels of sexual motivation, a new variable was created, named “lagged sexual motivation.” This variable was based on current sexual motivation by positioning the current sexual motivation score and the preceding, lagged, sexual motivation score in the same data row. Thus, the current score could be regressed on its preceding score. The resulting autoregressive or inertia coefficient usually varies between − 1 and 1 (Hamilton, 1994), which signifies that a person’s average level of sexual motivation hardly changes despite momentary fluctuations. If an individual’s sexual motivation inertia value is closer to 1, the resistance to change will be larger. For a value of 0.8 for instance, this would mean that the current level of sexual motivation will be less likely to change than for lower inertia values. In that case, a return to the average of momentary sexual motivation will happen less often and the next score of momentary sexual motivation is more likely to be similar to the previous score. Note that the first measurement scores of the lagged variable of each day were excluded from the analyses. Not doing so, would lead to a situation where the last measurement of the preceding day predicts the first measurement of the next day. This would lead to biased results, because the time lag in that case would be much larger than the average time lag of 90 min.

#### Positive Affect/Lagged Positive Affect

Positive affect was measured at beep level with 5 items using 7-point Likert-type scales. Items were adapted from the PANAS (Watson et al., 1988) and were worded as: “I feel…cheerful,” “…pleased,” “…happy,” “…relaxed” and “…enthusiastic.” For each person and each measurement, the scores on the 5 items were averaged to calculate the positive affect score for that specific beep measurement. Factor analysis on the current sample confirmed the 5 items as a separate factor (see above). McDonald’s (1999) ω was 0.83 at the person level and 0.93 at the beep level (Wiley, 2020). Lagged positive affect was constructed in the same way as lagged sexual motivation.

#### Negative Affect/Lagged Negative Affect

Negative affect was measured at beep level with 6 items using 7-point Likert-type scales (1 = “not at all” to 7 = “very much”). Items were adapted from the Positive Affect Negative Affect Scale (PANAS, Watson et al., 1988) and were worded as: “I feel…anxious,” “…guilty,” “…down,” “…lonely,” “…insecure” and “…irritated.” For each person and each measurement moment, the scores on the 6 items were averaged to calculate the negative affect score for that person at that specific moment. Factor analysis on the current sample confirmed the 6 items as constituting one factor (see above). McDonald’s ω (1999) was 0.90 at the person level and 0.60 at the beep level (Wiley, 2020). Lagged negative affect was constructed in the same way as lagged sexual motivation.

### Time-Invariant Variables

As person-level moderators we included three variables:

#### Sexual Excitation Proneness

Sexual excitation proneness (SES) was measured at the person level, using the SES subscale of the sexual inhibition/sexual excitation scales—short form (SIS/SES-SF; Carpenter et al., 2011). The complete SIS/SES-SF consists of 3 scales with 14 items in total. The SES subscale (6 items) is aimed at assessing sexual response in sexually arousing situations that do not involve a threat or risk. Confirmatory factor analysis on the current sample corroborated the expected three-factor structure of the SIS/SES-SF with the following fit measures: CFI = 0.98, TLI = 0.98, RSMEA = 0.06 and SRMR = 0.08. Items of the SES subscale were worded as: “When a sexually attractive stranger accidentally touches me, I easily become aroused.” Participants responded using 4-point Likert-type scales (1 = *“strongly disagree”* to 4 = *“strongly agree”).* SES showed high reliability; McDonalds’s ordinal *ω* was 0.89, as calculated with the R-package “userfriendlyscience” (Peters, 2018).

#### Sexual Inhibition Due to Threat of Performance Failure

Sexual inhibition, due to threat of performance failure, SIS1, was measured with 4 items of the SIS1 subscale of the SIS/SES-SF. The SIS1 subscale is aimed at assessing sexual response in sexually arousing situations that also involve the threat of performance failure. Failure might occur because one is easily distracted. Items of the SIS1 subscale were worded as: “I cannot get aroused unless I focus exclusively on sexual stimulation.” Participants responded using 4-point Likert-type scales (1 = *“strongly disagree”* to 4 = *“strongly agree”).* SIS1 showed sufficient reliability; McDonalds’s ordinal *ω* was 0.73 (Peters, 2018).

#### Sexual Inhibition Due to Threat of Performance Consequences

Sexual inhibition, due to threat of performance consequences (SIS2), was measured with 4 items of the SIS2 subscale of the SIS/SES-SF. The SIS2 subscale is aimed at assessing sexual response in sexually arousing situations that also involve the threat of performance consequences. Failure might occur due to the fear of being discovered while having sex and concerns about norms and values. Items of the SIS2 subscale were worded as: “If I can be seen by others while having sex, I am unlikely to stay sexually aroused.” Participants responded using a 4-point Likert-type scales (1 = *“strongly disagree”* to 4 = *“strongly agree”*)*.* SIS2 showed sufficient reliability; McDonalds’s ordinal *ω* was 0.74 (Peters, 2018).

We included six control variables: sexual frequency, depressive symptoms, anxiety, gender, age and relationship duration. Previous research has shown that these variables can impact sexual motivation and thus explain part of the variation in sexual motivation (Bancroft et al., 2003a; Dewitte & Mayer, 2018; Muise et al., 2019; Toates, 2014; Van Lankveld et al., 2018). They are included as person-level variables and this signals they will impact person-level averages of momentary sexual motivation.

#### Sexual Frequency

Sexual frequency represents the number of times the participant has been sexually active in the week that experience sampling data were collected. It was measured by counting the number of times unpartnered or partnered sexual activity was reported in the beep questionnaires. This means that sexual frequency has been measured at the beep level but that scores have been aggregated at the person level.

The Symptom Checklist—90—R (SCL-90-R) depressive symptoms subscale, consisting of 13 items, was used to assess relatively stable feelings of somberness and depression. Participants rated on a 5-point Likert-type scale how much they experienced symptoms in the past week (1—“*not at all*” to 5—“*a lot*”). Items were worded as: “Feeling hopeless about the future.” The items of the depressive symptoms subscale showed high internal consistency; McDonald’s (1999) ordinal ω was 0.87 (Peters, 2018).

The SCL-90-R anxiety subscale, consisting of 10 items, was used to assess relatively stable feelings of anxiety. Participants rated on a 5-point Likert-type scale how much they experienced symptoms in the past week (1—“*not at all*” to 5—“*a lot*”). Items were worded as: “Suddenly scared for no reason.” The items of the anxiety subscale showed high internal consistency; McDonald’s (1999) ordinal ω was 0.80 (Peters, 2018).

Gender (women/men), age (in years) and relationship duration (in years) were also included as control variables in the analyses.

### Data Analysis

Time-varying and time-invariant variables were analyzed using descriptive statistics. For this purpose, the beep-level variables were aggregated at the person-level to establish person mean values together with standard deviations and confidence intervals. Furthermore, the individual inertia effects of sexual motivation were assessed by analyzing the autoregressive models for each participant separately. In describing the sample, special attention was given to differences between women and men. *T* tests were used to assess these differences and effect sizes of the differences were added. We investigated if gender moderated the effects of mood on momentary sexual motivation. For this purpose, we performed separate preliminary multilevel analyses including only main effects and two-way interactions between beep-level variables and gender as predictors of momentary sexual motivation. Though gender differences were not of primary interest in this study, these analyses were added because gender differences in the effect of mood on momentary sexual motivation might well occur (Graham et al., 2004; Janssen et al., 2007). If gender would not prove to be a moderator in these preliminary analyses, it would not be included as a moderator in the main analyses thus limiting the complexity of the main analyses.

To analyze interindividual differences in intraindividual processes, multilevel analyses were applied. We investigated predictors of momentary sexual motivation, both with contemporaneous and lagged analyses (Epskamp et al., 2018). Multilevel analyses were needed because of the hierarchical structure of the data with multiple measurements per participant. Furthermore, multilevel models are flexible in handling missing data and do not require the number of observations per participants to be the same (Hox, Moerbeek & Van de Schoot, 2017).

In the basic multilevel model, a random intercept was included to account for differences in average sexual motivation levels between persons. The basic multilevel model was extended by adding beep-level predictors, negative and positive affect for the contemporaneous analyses and lagged negative affect, lagged positive affect (both cross-lagged effects) and lagged sexual motivation (the inertia effect) for the lagged analyses. All significant interactions between beep-level variables have been included as well. To test if the inertia effect varied between persons, the association between lagged sexual motivation and current sexual motivation was allowed to vary in the analyses. If the random-slopes model would improve the more basic model without random slopes, the conclusion would be warranted that there was considerable variability between persons in the inertia effect of sexual motivation (Aguinis et al., 2013; Bates et al., 2015). For the other beep-level variables, it was investigated as well if the model improved when their effects on sexual motivation were allowed to vary between persons. If the effects of beep-level predictors on sexual motivation would not vary significantly between persons, we would be able to conclude that these specific beep-level effects were similar for most individuals.

Next, person-level variables were added as predictors of sexual motivation, in both the contemporaneous and lagged analyses. As person-level characteristics, these variables need to be interpreted as predictors of the person-level averages of sexual motivation and not as predictors of beep-level scores. If the inclusion of random slopes for the beep-level effects (Hox et al., 2017, p. 21) leads to significant improvement of the models, cross-level interactions between person- and beep-level variables (Aguinis et al., 2013) will be investigated. These cross-level interactions allow for person-level predictors to be interpreted as moderators of beep-level processes (Kuppens et al., 2010).

The beep-level variables were person mean centered. Although this will result in a slightly downward bias in the estimates of the inertia effect, the estimates of the cross-level interactions will be least biased in this way (Hamaker & Grasman, 2015).

## Results

### Descriptives

Participants completed on average 53.8 (SD = 10.8) out of 70 beep questionnaires (76.8%). This is in accordance with compliance reported for ESM studies in general (Vachon et al., 2019). No participants completed less than 24 questionnaires. In total 87 women and 46 men participated; all participants were involved in steady heterosexual relationships for 6 months or longer and only one dyad was included in the sample. Two women and two men indicated to be exclusively homosexually oriented despite being involved in a heterosexual relationship. Participating women (*M* = 39.3; SD = 10.7) were on average younger than the men (M *M*46.4; SD = 11.4) in this study (*d* =  − 0.64; *p* < 0.001). Women’s momentary sexual motivation levels were on average 1.69 (SD = 0.80) and lower than men’s (*M* = 2.33; SD = 1.03) on a scale from 1 to 7 (*d* =  − 0.72; *p* < 0.001). Sexual motivation levels throughout the day started rising at beep 8. This pattern has been described more extensively in a previous study on these data (Van Lankveld et al., 2018). The frequency of solo and partnered sexual activity combined did not significantly differ for women and men (*d* = 0.14; *p* = 0.45); for this sample the average sexual frequency was around two times per week. Levels of sexual excitation and sexual inhibition proneness were similar to those found in a previous large-scale sample (Velten et al., 2018) and correlations between the three factors aligned with this previous result as well as did the differences found between women and men. The correlation between SES and SIS1 was −0.20 [95% CI: −0.36 to −0.03], between SES and SIS2: −0.29 [95% CI: −0.45 to −0.13], and between SIS1 and SIS2: 0.50 [95% CI: 0.37–0.62]. Women scored higher on SIS1 (*d* = 0.61; *p* = 0.001) and SIS2 (*d* = 0.52; *p* = 0.007) but lower on SES (*d* = −0.76; *p* < 0.001) than men. No significant differences for women and men were found for depressive symptoms or anxiety. The inertia of sexual motivation, as averaged across person level inertias, was 0.40 (SD = 0.32) and was not significantly different for women and men (*d* = −0.18; *p* = 0.36). Negative affect levels did not significantly differ between women and men (*d* = −0.08; *p* = 0.69), but positive affect levels were lower in women than in men (*d* = −0.43; *p* = 0.01).

Though not a primary target of this study, we investigated if associations between fluctuating positive and negative affect and momentary sexual motivation differed for women and men. Only gender and beep-level predictors of momentary sexual motivation were included in these analyses. For the contemporaneous model, we analyzed two-way cross-level interactions between positive affect, negative affect and gender predicting momentary sexual motivation and found that only the interaction between positive and negative affect was significant (estimate = −0.17; SE = 0.03; *p* < 0.001). For the lagged analyses, we analyzed two-way cross-level interactions between lagged positive affect, lagged negative affect, lagged sexual motivation and gender. We found that only the interaction between lagged sexual motivation and lagged negative affect was significant (estimate = −0.10; SE = 0.04; *p* = 0.026). Full results for both analyses are reported in Tables 2 and 3 in the supplementary material. Based on these results, we did not include gender as a moderator in the main analyses.

**Table 2:** Model with contemporaneous time-varying predictors and person-level predictors of momentary sexual motivation

Predictors	Estimate	SE	95% Confidence interval	*p* value
Fixed effects Intercept	1.21	0.56	0.10 to 2.31	.**031**
Negative affect^a^	−0.47	0.40	−1.27 to 0.33	.24
Positive affect^a^	0.08	0.32	−0.56 to 0.40	.79
Age	−0.01	0.01	−0.01 to 0.02	.41
Gender (female = 0; male = 1)	0.30	0.13	0.04 to 0.57	**.027**
Relationship duration	−0.01	0.01	−0.03 to 0.00	.12
Depressive symptoms	−0.02	0.01	−0.05 to 0.01	.22
Anxiety	0.02	0.03	−0.03 to 0.07	.43
Sexual frequency	0.14	0.03	0.08 to 0.20	** < .001**
SES	0.07	0.02	0.03 to 0.11	** < .001**
SIS1	−0.01	0.03	−0.08 to 0.06	.83
SIS2	−0.06	0.03	−0.12 to −0.00	**.042**
Negative affect*SES	0.03	0.02	0.00 to 0.06	**.047**
Negative affect*SIS1	−0.02	0.02	−0.07 to 0.03	.40
Negative affect*SIS2	0.02	0.02	−0.03 to 0.06	..42
Positive affect*SES	0.03	0.01	0.01 to 0.05	**.016**
Positive affect*SIS1	−0.00	0.02	−0.04 to 0.04	.91
Positive affect*SIS2	−0.00	0.02	−0.04 to 0.03	.82
Positive affect * Negative affect	−0.18	0.04	−0.25 to −0.11	** < .001**
Random effects Residual variance		0.818		
Random intercepts (variance in subject means of sexual motivation)		0.462		
Random slopes (positive affect)		0.141		
Random slopes (negative affect)		0.068		
ICC empty model		0.480		
Number of subjects/number of observations used		129/6866		

Bold values are statistically significant (*p* < .05)

^a^To assess the cross-level interactions between person-level variables and the time-varying variables with least amount of bias, the latter were person mean centered (Hamaker & Grasman, 2015)

**Table 3:** Model with lagged time-varying predictors and person level predictors of momentary sexual motivation

Predictors of sexual motivation	Estimate	SE	95% Confidence interval	*p* value
Fixed effects Intercept	1.14	0.58	− 0.00 to 2.30	**.045**
Lagged sexual motivation^a^ (inertia)	0.75	0.16	0.13 to 0.76	** < .001**
Lagged negative affect^a^	− 0.28	0.30	− 0.87 to 0.31	.35
Lagged positive affect^a^	0.03	0.02	− 0.01 to 0.07	.11
Age	0.01	0.01	− 0.01 to 0.02	.44
Gender (female = 0; male = 1)	0.35	0.14	0.07 to 0.64	**.016**
Relationship duration	– 0.01	0.01	− 0.03 to 0.00	.07
Depressive symptoms	− 0.00	0.02	− 0.04 to 0.03	.83
Anxiety	− 0.00	0.02	− 0.06 to 0.06	.99
Sexual frequency	0.19	0.03	0.13 to 0.26	** < .001**
SES	0.06	0.02	0.02 to 0.10	.**002**
SIS1	− 0.01	0.03	− 0.08 to 0.06	.83
SIS2	− 0.05	0.03	− 0.11 to 0.01	.07
Lagged negative affect*SES	0.03	0.01	0.01 to 0.05	**.025**
Lagged negative affect*SIS1	− 0.02	0.02	− 0.06 to 0.01	.15
Lagged negative affect*SIS2	0.01	0.02	− 0.02 to 0.05	.36
Lagged sexual motivation*SES	− 0.01	0.01	− 0.03 to 0.00	.17
Lagged sexual motivation*SIS1	− 0.03	0.02	− 0.07 to − 0.01	**.008**
Lagged sexual motivation*SIS2	0.03	0.01	− 0.00 to 0.05	.15
Lagged sexual motivation*Lagged negative affect	−0.09	0.04	− 0.16 to − 0.02	**.013**
Random effects (variance) Residual variance		0.695		
Random intercepts (variance in subject means of sexual motivation)		0.450		
Random slopes (lagged sexual motivation)		0.040		
Random slopes (lagged negative affect)		0.007		
ICC empty model		0.48		
Number of subjects/*N* observations used		129/5223		

Bold values are statistically significant (*p* < .05)

^a^To assess the cross-level interactions between person-level variables and time-varying variables, the latter have been person mean centered (Hamaker & Grasman, 2015)

### Momentary Sexual Motivation Predicted by Contemporaneous and Time-Invariant Variables

In Table 2, the results of the contemporaneous model are reported. Before this model was estimated, we established if the effect of negative or positive affect on momentary sexual motivation varied significantly between persons. After conducting model comparisons, we concluded that there was significant variability between persons in the effect of negative affect and positive affect on momentary sexual motivation. Therefore, we included the random-slopes variances for these two effects in the final model.

As shown in Table 2, there was no significant contemporaneous effect of negative (estimate = −0.47; SE = 0.040; *p* = 0.24) or positive affect (estimate = 0.08; SE = 0.32; *p* = 0.79) on momentary sexual motivation. However, a significant interaction effect of negative and positive affect on momentary sexual motivation was found (estimate = −0.18; SE = 0.04; *p* < 0.001). For higher values of negative affect, higher values of positive affect were associated with a less steep increase in momentary sexual motivation. For lower values of negative affect, higher values of positive affect were associated with a steeper increase of momentary sexual motivation.

Significant predictors of a person’s average sexual motivation were gender, sexual frequency, SES and SIS2 (see Table 2). Person-level averages of sexual motivation were 0.30 points lower for women than for men, on a scale from 1 to 7 (SE = 0.14; *p* = 0.027). An increase in sexual frequency of one partnered or solo sexual activity per week was associated with an increase in person-level average sexual motivation score of 0.14 points (SE = 0.03; *p* < 0.001). An increase in SES of 1 point (on a scale from 6 to 24) was associated with an increase in person-level average sexual motivation score of 0.07 points (SE = 0.02; *p* < 0.001). An increase in SIS2 of 1 point (on a scale from 4 to 16) was associated with a decrease in the person-level average sexual motivation score of 0.06 points (SD = 0.03; *p* = 0.047). There were no significant effects of SIS1, age, relationship duration, depressive symptoms or anxiety on the person-level averages of sexual motivation.

**Fig. 2:**
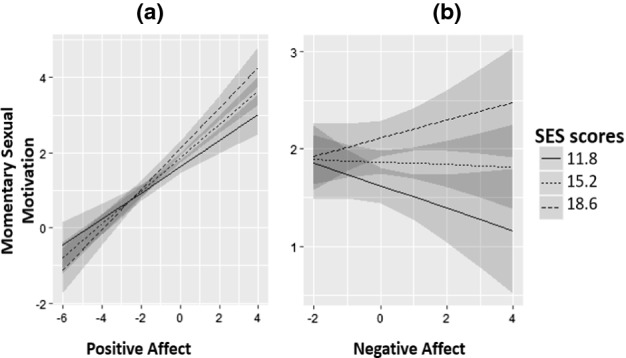
**a** Interaction between positive affect and SES predicting momentary sexual motivation. **b** Interaction between negative affect and SES predicting momentary sexual motivation. *Note* SES values refer to 1 standard deviation (SD) below the mean, the mean and 1 SD above the mean

Of the cross-level interactions of positive affect and SES, SIS1 and SIS2, only the interaction of positive affect and SES was significant (estimate = 0.03; SE = 0.01; *p* = 0.016). Figure 2a shows this interaction effect: when PA increased, momentary sexual motivation increased more in participants with higher SES scores than in participants with lower SES scores. The cross-level interactions of positive affect with SES, SIS1 and SIS2 decreased the random-slopes variance of positive affect from 0.083 to 0.068. This means that approximately 18% of the variance in the effect of positive affect on momentary sexual motivation was accounted for by SES, SIS1 and SIS2 (Aguinis et al., 2013).

Of the cross-level interactions of negative affect and SES, SIS1 or SIS2, only the interaction of negative affect and SES was significant (estimate = 0.03; SE = 0.02; *p* = 0.047). Figure 2b shows this interaction effect: an increase in negative affect was associated with an increase in momentary sexual motivation for participants more prone to sexual excitation. For lower SES levels, an increase in negative affect was not associated with a change in levels of momentary sexual motivation. Cross-level interactions of negative affect and SES, SIS1 and SIS2 decreased the random-slopes variance of negative affect from 0.151 to 0.141. This means that approximately 7% of the variance in the effect of negative affect on momentary sexual motivation was accounted for by SES, SIS1 and SIS2 (Aguinis et al., 2013). Follow-up analyses of a subsample of 46 participants (= 35%; 20 women/26 men) with SES scores of 17 or higher showed that an increase in negative affect of 1 is associated with an increase in momentary sexual motivation of 0.18 (*p* = 0.048) and that an increase in positive affect of 1 is associated with an increase in momentary sexual motivation levels of 0.51 (*p* < 0.001). Only significant predictors were included in these follow-up analyses and the only other significant predictor was sexual frequency (estimate = 0.26, *p* < 0.001).

### Momentary Sexual Motivation Predicted by Lagged and Time-invariant Variables

Table 3 shows the results for the lagged model. Before this model was estimated, we determined if the effect of lagged sexual motivation, lagged negative affect and lagged positive affect on current sexual motivation varied significantly between persons. After conducting model comparisons, we concluded that there was significant variability between individuals in the effect of lagged negative affect and lagged sexual motivation on current sexual motivation and we included the random slopes for these two effects in the final model. No significant variability was found for the effect of lagged positive affect on sexual motivation. Therefore, we did not add the random slopes for lagged positive affect to the lagged model.

As Table 3 shows, lagged sexual motivation was a significant predictor of current sexual motivation. The estimate of the inertia effect was 0.49 (SE = 0.04; *p* < 0.001). No significant effects of lagged negative affect (estimate = −0.30; SE = 0.30; *p* = 0.31), or lagged positive affect (estimate = 0.03; SE = 0.02; *p* = 0.13) on current sexual motivation were found. However, a significant interaction of lagged negative affect and lagged sexual motivation on current sexual motivation was found (estimate = −0.18; SE = 0.04; *p* < 0.001). For lower scores of lagged negative affect, the association between lagged sexual motivation and current sexual motivation was more positive than for higher lagged negative affect scores. This means that high negative affect dampened the positive association of lagged and current sexual motivation more than low negative affect. Gender, sexual frequency, SES and SIS2 were significant predictors of the person level averages of sexual motivation (see Table 3). The results for the main effects of all person-level variables were comparable to the results reported for the contemporaneous model in Table 2.

**Fig. 3:**
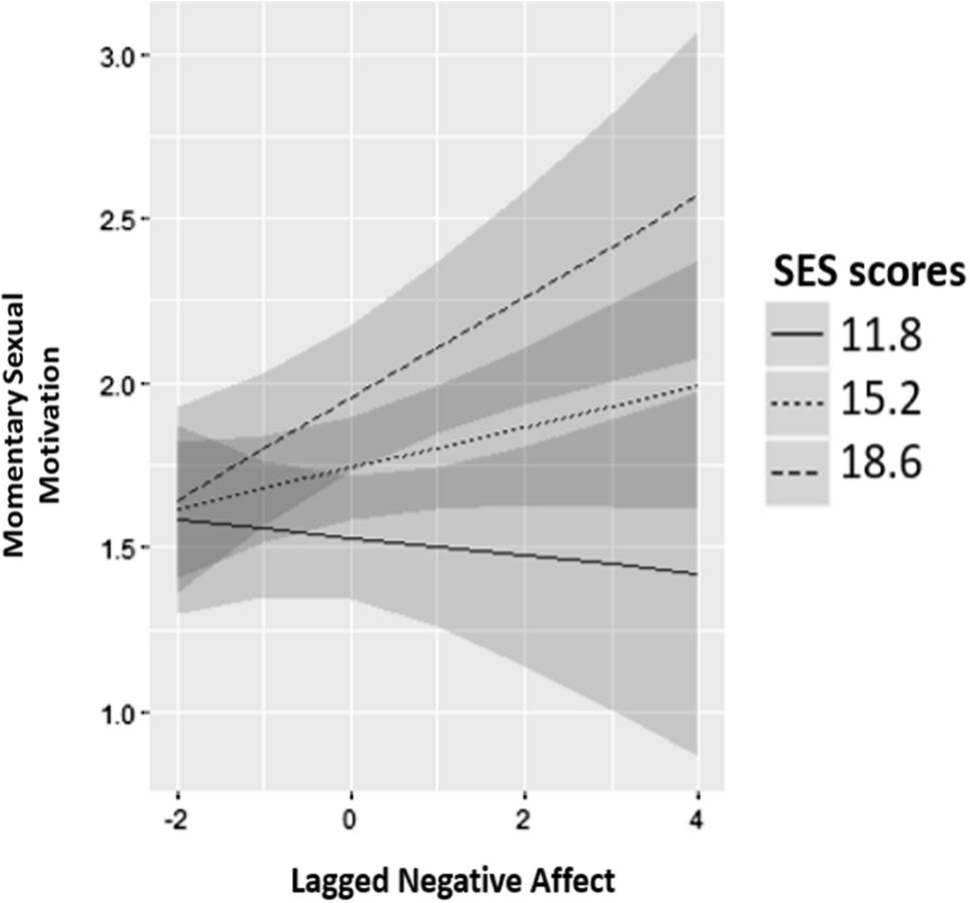
Interaction of lagged negative affect and SES in predicting momentary sexual motivation. SES values refer to 1 SD below the mean, the mean and 1 SD above the mean

Of the cross-level interactions of lagged negative affect and SES, SIS1 and SIS2, only the interaction with SES was significant (estimate = 0.03; SE = 0.01; *p* = 0.016). Figure 3 shows this interaction effect: an increase in lagged negative affect forecasted an increase in current momentary sexual motivation for higher SES levels. An increase in lagged negative affect was not associated with a change in levels of current momentary sexual motivation for lower SES levels. Cross-level interactions of lagged negative affect and SES, SIS1 and SIS2 decreased the random-slopes variance of lagged negative affect from 0.010 to 0.007. This means that approximately 30% of the variance in the effect of lagged negative affect on current momentary sexual motivation was accounted for by SES, SIS1 and SIS2 (Aguinis et al., 2013). Follow-up analyses of a subsample of 46 participants (= 35%; 20 women/26 men) with SES scores of 17 or higher showed that an increase in lagged negative affect of 1 forecasted an increase in current momentary sexual motivation of 0.18 (SE = 0.07, *p* = 0.010). Only significant predictors were included in these follow-up analyses and the other significant predictors were sexual frequency (estimate = 0.31, SE = 0.05, *p* < 0.001) and lagged sexual desire (estimate = 0.45, SE = 0.04, *p* < 0.001).

The cross-level interaction of lagged sexual motivation (the autoregressive or inertia effect) with SIS1 was significant (estimate = −0.03, SE = 0.01, *p* = 0.008); the interaction of lagged sexual motivation with SIS2 was not significant (estimate = 0.03, SE = 0.01, *p* = 0.15). The two interactions seemed to diverge: sexual inhibition due to threat of performance failure was negatively associated with the inertia of sexual motivation while sexual inhibition due to threat of performance consequences was positively, though nonsignificantly, associated with the inertia of sexual motivation. No significant cross-level interaction effect was found for lagged sexual motivation and SES (estimate = −0.01, SE = 0.01, *p* = 0.16). Cross-level interactions of lagged sexual motivation and SES, SIS1 and SIS2 decreased the random-slopes variance of lagged sexual desire from 0.044 to 0.040. This means that approximately 9% of the variance of the inertia effect of sexual motivation was accounted for by SES, SIS1 and SIS2 (Aguinis et al., 2013).

## Discussion

The aim of this study was to investigate the associations between hour-to-hour fluctuations in mood and momentary sexual motivation, and to assess if the three factors of the dual control model moderated these associations. The large number of measurements per participant within a relatively short period of time allowed for the analyses of both contemporaneous and short-term lagged associations. Our hypotheses regarding the associations of mood and momentary sexual motivation, and the moderation of these associations by sexual excitation and sexual inhibition proneness, were partly confirmed. Before discussing our findings, we note that there were no differences between women and men in the associations between mood and momentary sexual motivation. Though relevant moderators of sex differences might still exist, our results implicated that in general women and men show large similarities in the impact of mood on momentary sexual motivation.

### Positive Affect, Momentary Sexual Motivation, and the Dual Control Model

With regard to positive affect, the final contemporaneous and lagged models showed no significant association between positive affect and momentary sexual motivation. However, a significant interaction between current positive and negative affect was found in the contemporaneous model, which implied that for higher levels of current negative affect an increase in current positive affect is associated with a less steep increase in momentary sexual motivation. Thus, negative affect can be seen as dampening the positive impact of positive mood on sexual motivation. Both for high and low values of negative affect, there was a positive association between positive affect and momentary sexual motivation, indicating that positive mood in general is associated with higher levels of sexual desire, subjective sexual arousal and openness to sex.

The contemporaneous analyses also showed significant variability between individuals in the effect of positive mood on momentary sexual motivation and this effect was moderated by one of the factors of the DCM: positive affect had a stronger positive association with momentary sexual motivation for participants more prone to sexual excitation. This confirmed our hypothesis and suggests that people who are more easily sexually excited might experience more positive feelings related to momentary increases in sexual motivation.

For the lagged analyses, and contrary to our expectations, no significant variability in the effect of lagged positive affect on current sexual motivation was found. Because lagged positive affect also did not predict sexual motivation, we may assume that the effect of preceding positive affect on current sexual motivation is similar and absent for most individuals, which implies that on an hour-to-hour basis positive affect does not forecast sexual motivation. This result for the lagged analyses precludes the testing of moderating effects (Hox et al., 2017) of DCM factors on associations between lagged positive affect and current sexual motivation.

We found no indications that sexual motivation will, for part of the population, decrease when in positive mood states, as found by some (Janssen et al., 2013). Our results also seem to diverge from Kalmbach and Pillai’s daily diary study (2014), which showed that higher joviality (“happy,” Kalmbach & Pillai, 2014, p. 2941) did forecast higher sexual desire the next day. Combining their results with ours might implicate that positive affect forecasts sexual motivation on a day-to-day basis, but that there is no hour-to-hour effect.

To conclude this section on positive affect and sexual motivation: we did not find a moderating effect of sexual inhibition measures on associations between positive affect and momentary sexual motivation. The contemporaneous positive association of positive affect and sexual motivation indicated that an upregulation of momentary sexual motivation in general goes together with positive and pleasurable mood states, and mostly so for people with elevated levels of sexual excitation proneness. For this group, it seems there is a stronger positive connection between fluctuations in positive affect and momentary sexual motivation, suggesting that this connection is more easily accessible for them and that they can adapt to positive mood states more quickly by adjusting their levels of momentary sexual motivation.

### Negative Affect, Momentary Sexual Motivation, and the Dual Control Model

With regard to negative affect, the final models showed no significant associations between current or lagged negative affect and momentary sexual motivation. However, a significant interaction between negative and positive affect was found, as discussed above. Furthermore, we found considerable individual variability both for the contemporaneous and lagged associations of negative affect and momentary sexual motivation. This variability allowed for investigations of the moderation of beep-level effects by person-level factors of the DCM. Our findings showed that sexual excitation proneness moderated the association between current or lagged negative affect and momentary sexual motivation. For individuals with above-average levels of sexual excitation proneness, higher current as well as lagged negative affect were associated with higher levels of momentary sexual motivation. There was no association between current or lagged negative affect and momentary sexual motivation for average or below average levels of sexual excitation proneness.

Our results are in line with previous results of cross-sectional research (Bancroft et al, 2003a; Lykins et al., 2006): for part of the population sexual interest increases in negative mood states, in particular for individuals with higher levels of sexual excitation proneness. We had not expected to corroborate these results due to the characteristics of our sample. Contrary to the sample of Bancroft et al. (2003a), the current sample consisted of both women and men (as opposed to only men), with a relatively high mean age (42 vs. 28 years) and with all participants involved in long-term relationships. Also, the current sample showed rather low frequencies of sexual activity (around 2 sexual activities per week), if compared to, for instance, a recent large-scale Canadian study in which a weekly average of 6.2 sexual activities was reported (Kingston et al., 2020). Nonetheless, our results suggest that also for part of this sample of relatively older women and men in long-term relationships, increases in current or lagged negative affect were associated with increases in momentary sexual motivation.

Emotion regulation mechanisms in which sexual urges and behaviors serve to deflect negative mood states, have previously been presented as characteristics of dysregulated sexuality (Carnes, 1983; Goodman, 1992; Kafka, 2010) and have been described as “sexually acting out” (Bancroft & Vukadinovic, 2004, p. 228). However, as our research suggests, the pattern to deflect negative mood by higher motivation to become sexual might not be limited to out-of-control sexuality. It might constitute a common and non-pathological mechanism of sexual and emotional self-regulation that is not necessarily problematic. It is not clear whether momentary sexual motivation is deliberately used with the intention to downregulate negative affect or if this process occurs outside of conscious emotion regulation. The suggestion that the positive association between negative mood and sexual motivation is a barrier to establishing relationships (Bancroft et al., 2004) is not supported by the current study. However, a more prominent positive association between negative affect and momentary sexual motivation might still constitute an important characteristic of problematic hyper- or hyposexuality (Gilliland et al., 2011; Walton et al., 2017). Further research into the fluctuations of mood and sexual motivation is, we believe, required to assess the impact of mood on sexuality in hyper- and hyposexual populations.

With regard to the combined effects of positive and negative affect on momentary sexual motivation, our results suggest that specifically for individuals with higher sexual excitation proneness, an increase in both positive and negative affect is associated with elevated momentary sexual motivation. This seems to suggest that for this group, “to be in the mood” does not only mean to be in a “good” mood (e.g., cheerful, happy, etc.) but can also mean to be in “bad” mood (e.g., down, insecure, etc.). The similar effects on sexual motivation of apparently opposite emotions are in accordance with previous findings implicating that ambivalent affect or “being in any mood” (Janssen et al., 2013, p.683) is positively associated with elevated levels of sexual desire (Peterson & Janssen, 2007) or with the probability to masturbate or watch porn (Miner et al., 2019). The results for ambivalent affect were not replicated in the lagged analyses: only lagged negative affect forecasted momentary sexual motivation and only for people with higher SES scores. This suggests that only lagged negative affect, and not lagged positive affect or a combination of both, will have a temporal effect on sexual motivation for people more prone to sexual excitation.

### The Inertia of Sexual Motivation

We investigated the autoregressive effect or inertia of sexual motivation and found that preceding sexual motivation is a strong predictor of current sexual motivation. Given an average time lag between measurements of 90 min, our results implicated that for people in steady relationships, sexual motivation often lingered for longer than one time lag. This does not imply that sexual motivation will stay exactly the same, but it seems to mark that if a person’s current sexual motivation is higher than average, it will probably be above average as well in a few hours from now.

We explored associations between the inertia of sexual motivation and sexual excitation and inhibition proneness. Our results showed that only sexual inhibition due to threat of performance failure (SIS1) impacted the inertia of sexual motivation. SIS1 was negatively associated with the inertia of sexual motivation, indicating that more frequent fluctuations in sexual motivation were associated with higher person levels of inhibition due to the threat of performance failure. As SIS1 did not impact average sexual motivation levels, these results seem to suggest that the construct of SIS1 might actually represent a mixture of inhibitory and excitatory response patterns. People with higher SIS1 scores might experience stronger sexual inhibition (question 12 and 13 of the SIS/SES-SF SIS1 scale, Carpenter et al., 2011, p. 238), but react to this loss of sexual excitation by consciously taking action to stay excited (question 4 and 9 of the SIS/SES-SF SIS1 scale, Carpenter et al., 2011, p. 238). When inhibition leads to downregulation of sexual motivation, conscious effort is taken to upregulate sexual motivation. What thus could ensue is a higher frequency of fluctuations in sexual motivation, which might explain the lower sexual motivation inertia scores for people with higher SIS1 levels.

These results with regard to sexual motivation inertia and our explanations thereof, are rather exploratory as there has been no preceding research on this subject. Notwithstanding, our results do indicate that the concept of sexual motivation inertia can be used to explain processes involving momentary sexual motivation. This might be particularly useful in hypo- or hypersexual subpopulations where sexual motivation inertia might be able to explain differences in the down- or upregulation of sexual desire, subjective sexual arousal and openness to sex. More generally, our results suggest that momentary sexual motivation might behave differently from positive of negative affect, but can nevertheless be seen as an emotional state, ruled by the same emotion regulatory principles of down- and upregulation and resistance to change (Kuppens & Verduyn, 2017), and our study illustrates how sexual motivation can be investigated focusing on these principles.

### Limitations

We wish to acknowledge several limitations of this study. As a general limitation, the effect sizes for significant effects were often small, especially for the interaction effects, and replication research should be undertaken to test if our results hold (Lakens & Evers, 2014). Furthermore, the application of snowball sampling by master students might have resulted in a more highly educated sample compared to the general population. The diversity of the sample was limited as only participants involved in heterosexual relationships were recruited (with 3% indicating to be exclusively homosexual) and most participants self-identified as Caucasian. Further research needs to be more inclusive with regard to sexual orientation and ethnicity in order to allow for generalization of results to a broader population. Future studies should preferably also include samples from non-western countries.

With regard to the application of ILD, we mention that ILD offers researchers new possibilities but can also pose specific problems. The burden of participating in ILD can be substantial (Van Genugten et al., 2020) and can lead to bias in response patterns (Rintala et al., 2020). However, reported bias is generally small and depends more on questionnaire length than on beep frequency (De Vuyst et al., 2019). As the beep questionnaire used in this study was relatively short, and also because compliance in this study has been high, we can assume that bias in response patterns was limited.

With regard to data analysis, it has been noted (Peterson & Janssen, 2007) that studying the separate emotions that constitute positive and negative affect might improve our understanding of the associations between mood and sexuality. Although we acknowledge this, we have not analyzed the different emotions separately in the current study due to the extended reporting these analyses would require. Furthermore, also due to the need to limit ourselves, we did not differentiate between partnered and solo sexual activities, though the role of partner relations could further elucidate the complex associations between mood and sexuality (Dewitte & Mayer, 2018; Muise et al., 2019).

We like to stress that unknown confounders and predictors might still exist. A possible confounder for the current analysis could have been fluctuating self-esteem which has not been investigated in this study or elsewhere in the context of ILD and sexual motivation. However, self-esteem might significantly impact sexual motivation, as has been suggested by therapists (Kisjes & Kruk, 2021; Van Zessen, 2009).

With regard to gender differences in the associations between mood and momentary sexual motivation, we wish to mention that this subject might be investigated more deeply than we did in this study, for instance, DCM factors as moderators of gender differences.

Finally, we mention that we have only investigated the univariate model with momentary sexual motivation as an outcome variable. A bi- or multivariate model, including momentary sexual motivation as predictor and positive or negative affect as outcome, might have been able to provide more detail to the intricate and possibly circular associations of mood states and momentary sexual motivation. As we have made our data available, such models might be investigated in the future by others. With regard to datasets collected with ILD, we like to state that no single study can hope to capture and convey the complete information that such datasets contain.

### Conclusion

Despite these limitations, this study has shown the potential of ILD methods to investigate the hour-to-hour interplay of fluctuating mood states and momentary sexual motivation. Measures conceptualized within the framework of the DCM proved to be viable moderators of these dynamic processes. Our results suggest that mood states have an impact on momentary sexual motivation specifically for people with higher levels of sexual excitation proneness. In particular, we found that higher negative affect and higher positive affect were associated with elevated momentary sexual motivation for this group, but that for them only higher lagged negative affect forecasted higher sexual motivation. Our results also implicated that sexual motivation can be viewed as inert, that is, within a 90-min window, momentary sexual motivation in general remains relatively stable. Individual differences in the inertia of sexual motivation were impacted by sexual inhibition proneness due to threat of performance failure as conceptualized by the DCM. Previously, there has been a call for a more central position of emotion research within sex research (Everaerd et al., 2006), which we underwrite, and we recommend that more ILD research into mood and sexual motivation is undertaken. Sexual feelings and consequent sexual behavior exist within the total spectrum of our emotional experiences and are intricately related to emotions in general. Research into the nuances of mood and sexuality can profit, we believe, from low-intrusive and ecologically valid methods that take the fluctuating nature of mood states and sexual feelings into account.

## Supplementary Information

Below is the link to the electronic supplementary material.

Supplementary file1 (DOCX 24 kb)

## Data Availability

Raw data and R-code used to analyze the data are stored on the Open Science Framework and are available via https://osf.io/3yfbx/?view_only=07f3d4c2b100466f815a5f8bc1b4ac6d.
