# Correction to: The Relation of Mood and Sexual Desire: An Experience Sampling Perspective on the Dual Control Model

**DOI:** 10.1007/s10508-022-02415-3

**Published:** 2022-09-06

**Authors:** Piet van Tuijl, Peter Verboon, Jacques van Lankveld

**Affiliations:** 1 Department of Psychology, Open Universiteit, 6419 Heerlen, The Netherlands; 2 Egelantierstraat 138, 3551GG Utrecht, Netherlands

## Correction to: Archives of Sexual Behavior 10.1007/s10508-022-02357-w

Placeholders in the form “xxx” erroneously appeared in the **Sample and Procedure** and **Descriptives** sections in this article as originally published.

The original article has been corrected.

