# Supplementary material for: The Relation of Mood and Sexual Desire: An Experience Sampling Perspective on the Dual Control Model
Source: Arch Sex Behav. 2022 Jul 27;51(8):3871–86. doi: 10.1007/s10508-022-02357-w (PMC9663403; doi:10.1007/s10508-022-02357-w)
Supplement: Supplementary file 1 — Supplementary file1 (DOCX 24 kb) [file 10508_2022_2357_MOESM1_ESM.docx]

**Supplementary Material to “The relation between mood and momentary sexual motivation: An experience sampling perspective on the Dual Control Model”**

Table 1

*Confirmatory Factor Analysis of Momentary Sexual Motivation, Positive Affect and Negative Affect (factor loadings).*

| Beep-level items | Factor loadings beep-level (SE) | Factor loadings person-level (SE) |
| --- | --- | --- |
| ***Momentary Sexual Motivation*** |  |  |
| Sexual Desire | 1 | 1 |
| Subjective Sexual Arousal | 0.83 (0.01) | 0.82 (0.03) |
| Openness to Sexual Contact | 1.10 (0.01) | 1.08 (0.05) |
| ***Positive Affect*** |  |  |
| Cheerful | 1 | 1 |
| Pleased | 1.03 (0.02) | 1.07 (0.05) |
| Happy | 0.94 (0.02) | 1.08 (0.06) |
| Relaxed | 1.04 (0.02) | 0.99 (0.08) |
| Enthusiastic | 1.02 (0.02) | 1.27 (0.10) |
| ***Negative Affect*** |  |  |
| Lonely | 1 | 1 |
| Anxious | 0.65 (0.03) | 1.16 (0.10) |
| Down | 2.10 (0.07) | 1.30 (0.17) |
| Insecure | 1.27 (0.06) | 1.88 (0.17) |
| Guilty | 0.72 (0.04) | 1.43 (0.14) |
| Irritated | 2.40 (0.10) | 1.47 (0.19) |

^Fit measures for the CFA have been reported in the main article.^

Table 2

*Model with contemporaneous time-varying predictors and gender predicting momentary sexual motivation.*

| Predictors | *Estimate* | *SE* | *95% Confidence Interval* | *p-value* |
| --- | --- | --- | --- | --- |
| **Fixed Effects**  Intercept | 1.67 | 0.09 | 1.49 – 1.75 | **<.001** |
| Negative Affect^1^ | -0.00 | 0.06 | -0.12 – 0.12 | .97 |
| Positive Affect^1^ | 0.43 | 0.05 | 0.33 – 0.53 | **<.001** |
| Gender (Female = 0; Male = 1) | 0.64 | 0.16 | 0.32 – 0.96 | **<.001** |
| Negatieve Affect * Positive Affect | -0.17 | 0.03 | -0.24 - -0.10 | **<.001** |
| Negative Affect * Gender | -0.01 | 0.10 | -0.21 – 0.19 | .89 |
| Positive Affect * Gender | 0.04 | 0.08 | -0.13 – 0.21 | .64 |
| **Random Effects**  Residual variance |  | 0.83 |  |  |
| Random intercepts (variance in  subject means of Momentary Sexual Motivation) |  | 0.76 |  |  |
| Random slopes (Positive Affect) |  | 0.16 |  |  |
| Random slopes (Negative Affect) |  | 0.07 |  |  |
| Number of subjects / Number of observations used |  | 133 / 7021 |  |  |

^1To assess the cross-level interactions between person level variables and the time-varying variables, the latter were person mean centered (Hamaker and Grasman, 2014).^

Table 3

*Model with lagged time-varying predictors and gender predicting momentary sexual motivation.*

| Predictors | *Estimate* | *SE* | *95% Confidence Interval* | *p-value* |
| --- | --- | --- | --- | --- |
| **Fixed Effects**  Intercept | 1.67 | 0.10 | 1.48 – 1.86 | **<.001** |
| Lagged Sexual Motivation | 0.42 | 0.03 | 0.36 – 0.49 | **<.001** |
| Lagged Negative Affect^1^ | 0.09 | 0.05 | -0.01 – 0.19 | .09 |
| Lagged Positive Affect^1^ | 0.03 | 0.02 | -0.01 – 0.07 | .14 |
| Gender (Female = 0; Male = 1) | 0.62 | 0.16 | 0.30 – 0.94 | **<.001** |
| Lagged NA* Lagged PA | 0.02 | 0.04 | -0.05 – 0.09 | .51 |
| Lagged NA* Lagged SM | -0.10 | 0.04 | -0.18 - -0.01 | **.026** |
| Lagged PA * Lagged SM | 0.00 | 0.02 | -0.04 – 0.04 | .87 |
| Lagged SM * Gender | 0.02 | 0.05 | -0.08 – 0.12 | .81 |
| Lagged NA* Gender | -0.04 | 0.09 | -0.21 – 0.14 | .66 |
| Lagged PA * Gender | -0.02 | 0.04 | -0.11 – 0.06 | .55 |
| **Random Effects**  Residual variance |  | 0.70 |  |  |
| Random intercepts (variance in  subject means of Momentary Sexual Motivation) |  | 0.77 |  |  |
| Random slopes (Lagged Sexual Motivation) |  | 0.05 |  |  |
| Random slopes (Lagged Negative Affect)^2^ |  | 0.02 |  |  |
| Number of subjects / Number of observations used |  | 133 / 5339 |  |  |

^1To assess the cross-level interactions between person level variables and the time-varying variables, the latter have been person mean centered (Hamaker and Grasman, 2014).^

^2The random slopes of positive affect were not added, as adding them did not sufficiently improve the model.^
